# Population Genetic Differences Between Two Cucurbit Leaf Beetles With Contrasting Feeding Preferences

**DOI:** 10.1002/ece3.73543

**Published:** 2026-04-20

**Authors:** Bo He, Mingkang Xiao, Tianyu Ye, Gonghua Lin, Fang Zhao, Liancheng Liu, Huanhuan Li, Tianjuan Su, Zuhao Huang

**Affiliations:** ^1^ School of Life Sciences, Key Laboratory of Jiangxi Province for Biological Invasion and Biosecurity Jinggangshan University Ji'an China

**Keywords:** agriculture pest, cucurbit leaf beetles, feeding difference, mitochondrial genome, population genetics

## Abstract

The *Aulacophora* beetles are pests closely associated with cucurbit plants. However, research on the fine‐scale population genetics of species within this genus remains limited. This study employed mitogenome data from 211 individuals (102 *A. indica* and 109 *A. lewisii* ) to examine the population genetic patterns of these two common cucurbit pests, which exhibit distinct dietary preferences ( *A. indica* and *A. lewisii* ). Our analysis revealed that the polyphagous *A. indica* exhibited significantly higher genetic diversity than the oligophagous *A. lewisii* (*p* < 0.001). Populations of *A. indica* showed lower genetic differentiation (*F*_ST_ = 0.089) and higher gene flow (*N*_m_ = 212.901), with no correlation between genetic differentiation and geographic isolation (*p* = 0.587). In contrast, populations of *A. lewisii* displayed higher genetic differentiation (*F*_ST_ = 0.186) and lower gene flow (*N*_m_ = 62.860), consistent with the isolation by distance (IBD) model (*p* < 0.001). These significant differences suggest that dietary breadth may play a key role in shaping population genetic characteristics. Neutrality tests revealed signals consistent with either recent population expansion or positive selection in both species, suggesting that human‐mediated crop cultivation may have influenced their genetic diversity and structure.

## Graphical abstract

The study revealed that the polyphagous *A. indica* exhibits higher genetic diversity, lower differentiation, and higher gene flow than the oligophagous *A. lewisii* , with these differences attributed to their distinct dietary preferences. *A. lewisii* populations showed significant isolation‐by‐distance, whereas *A. indica* did not, indicating different evolutionary patterns. Neutrality tests revealed signals consistent with either recent population expansion or positive selection in both species, suggesting that human‐mediated crop cultivation may have influenced their genetic diversity and structure.

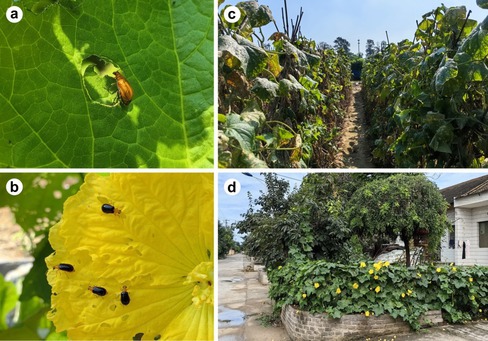

## 1 Introduction

Insect pests show increased damage due to the changes in environment, management procedures, or genetic adaptions to local environments (Hoffmann and Sgro 2011; Riegler 2018). Furthermore, direct effects of climate change and management practices, as well as the evolution of pesticide resistance (Bergé and Ricroch 2010; Nauen et al. 2019), may directly or indirectly contribute to the dispersal of species. Contemporary factors, including human‐mediated crop cultivation, can also strongly influence geographic distribution and genetic variation within populations (Bradshaw et al. 2016; Cao et al. 2017, 2021; Drahun et al. 2021; Simberloff et al. 2013; Xu et al. 2022). Therefore, studies on patterns of population genetic differentiation and connectivity between populations would help to determine how past and present factors contribute to population genetic patterns, and accordingly provide a deeper understanding of species evolutionary history and ecological adaptation, which can help develop appropriate conservation and management strategies (Drahun et al. 2021; Xu et al. 2022; Ye et al. 2022).

The genus *Aulacophora* (Coleoptera: Chrysomelidae) is a group of pests that harm a wide variety of monocotyledonous and dicotyledonous plants, particularly cucurbits (Cucurbitaceae) (Aslan et al. 2000; Lewis and Metcalf 1996). The organs of cucurbit plants, such as the leaves, often contain cucurbitacins, a group of oxygenated tetracyclic triterpenoids that are highly toxic to humans and most animals (Gorski et al. 1985; Kamata et al. 2023). In contrast, cucurbitacins act as a feeding stimulant for *Aulacophora* beetles, promoting the specific feeding of cucurbit plants by these beetles (Abe et al. 2000; Abe and Matsuda 2005; Kong et al. 2004), which are commonly referred to as cucurbit leaf beetles. Their larvae feed on roots and underground stems (Ahmad et al. 2013), while adults are voracious feeders of leaves, seedlings, flowers, and fruits (Butani and Jotwani 1984; Hassan et al. 2012). They usually occur in large numbers, substantially reducing the crop yields. Three species— *A. indica* , *A. lewisii* , and *A. foveicollis* —are major pests, collectively endanger over 81 cucurbit plant species and cause 30%~100% yield losses (Kamal et al. 2014; Rashid et al. 2014; Reeta and Johri 2003). Although *A. foveicollis* is also a destructive pest, the present study focuses on *A. indica* and *A. lewisii* due to their widespread distribution and contrasting dietary preferences.

**FIGURE 1:**
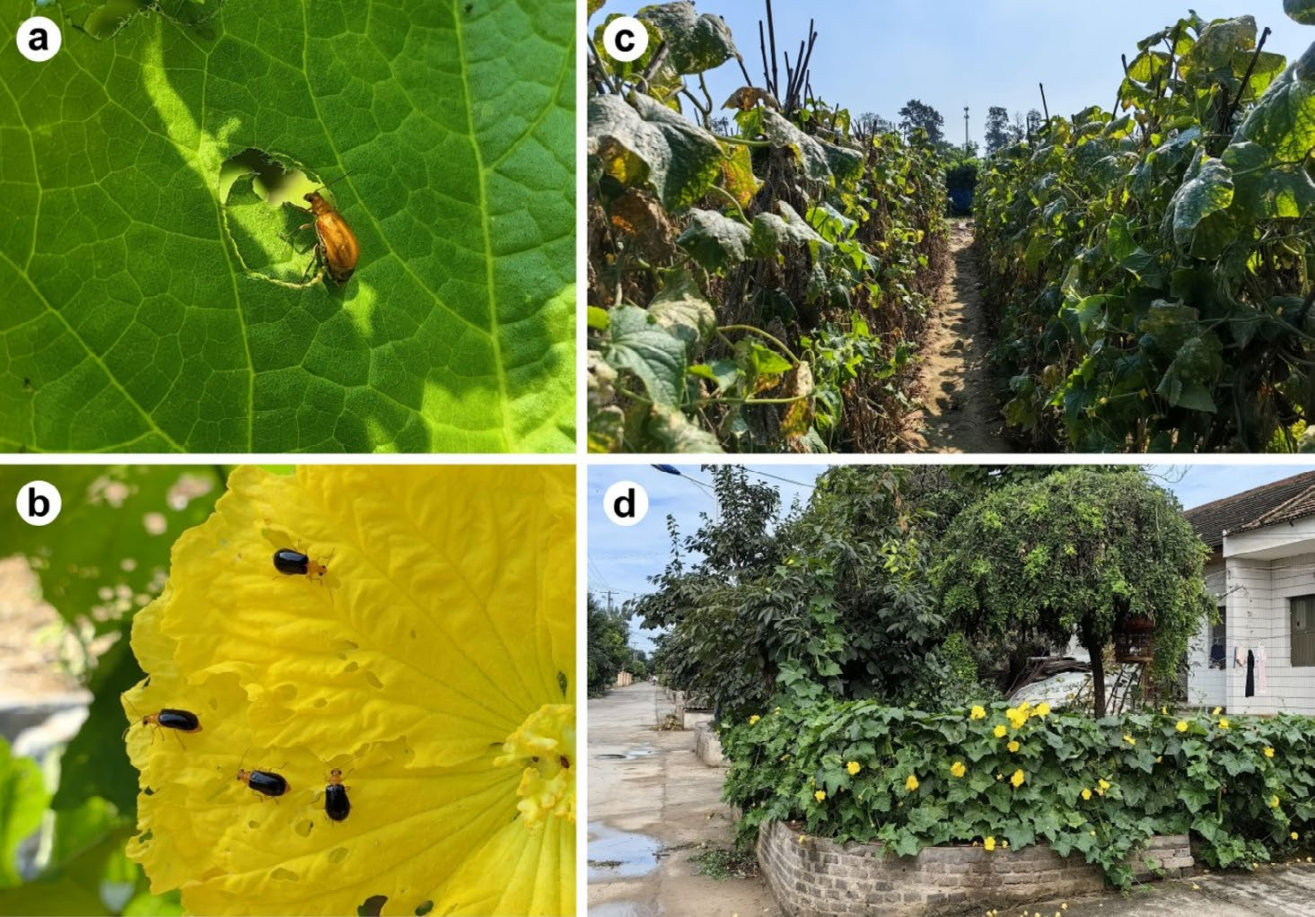
The damage caused by cucurbit leaf beetles on host plants and their living environment. (a) The *A. indica* feeds on loofah leaves. During the feeding process, it bites a ring around the feeding area to inhibit the synthesis of cucurbitacin and then eats the leaf tissue inside; (b) The *A. lewisii* is feeding on the petals of loofah; (c) *Aulacophora indica* and *A. lewisii* seek food and complete their life cycles in these environments; (d) loofah grown at the entrance of homes in rural China.

*Aulacophora indica* and *A. lewisii* are two common cucurbit pests with contrasting dietary preferences. The former is polyphagous, feeding on a wide range of cucurbits including cucumber, loofah, white gourd, sweet gourd, bottle gourd, watermelon, muskmelon, and many other crops (Kamal et al. 2014; Lee and Beenen 2015). When feeding on leaves, adults create irregular holes that impair photosynthesis (Figure 1a). In contrast, *A. lewisii* is oligophagous, feeding on fewer plant species (Figure 1b), mainly loofah, and occasionally bitter gourd (Das et al. 2024; Sarker et al. 2019). Although the two species are closely related and widely distributed across tropical and subtropical Asia, their population genetic variation remains largely unknown.

Understanding population genetic structure is essential for pest management, as it reveals patterns of dispersal, gene flow, and adaptive potential. Populations with high genetic diversity and connectivity may exhibit greater resilience and rapid adaptation to control measures (Beaurepaire et al. 2024; Endersby et al. 2011), whereas isolated populations may be more susceptible to local extinction but also may develop localized resistance (Miller et al. 2005; Zheng et al. 2015). Population genetic analyses can also identify sources of outbreak populations, track invasion pathways, and inform the design of area‐wide management strategies (Cao et al. 2017; Xu et al. 2022). Given the economic importance of *Aulacophora* pests, characterizing their genetic diversity, differentiation, and demographic history is crucial for predicting their adaptive potential and developing sustainable control strategies.

In the present study, we conducted a distribution survey and sampled from multiple geographical areas of China. High throughput sequencing was used to obtain 13 protein‐coding genes (PCGs) of 211 mitochondrial genomes representing 15 geographical populations for each of *A. indica* and *A. lewisii* . For these two species, we examined their genetic diversity, population structure, and population dynamic history. We tested the hypothesis that the spatial genetic structure of the two co‐distributed species of *Aulacophora* pests would be different. The information can be used to analyze population evolution and further assess its ecological fitness. We then analyzed the driving factors that influence the differences in population genetic structure. The results obtained in this paper contribute to our understanding of the evolutionary origin of these two pests, which provide a useful theoretical foundation for developing appropriate pest control strategies.

## 2 Materials and Methods

### 2.1 Sample Collection and DNA Extraction

**FIGURE 2:**
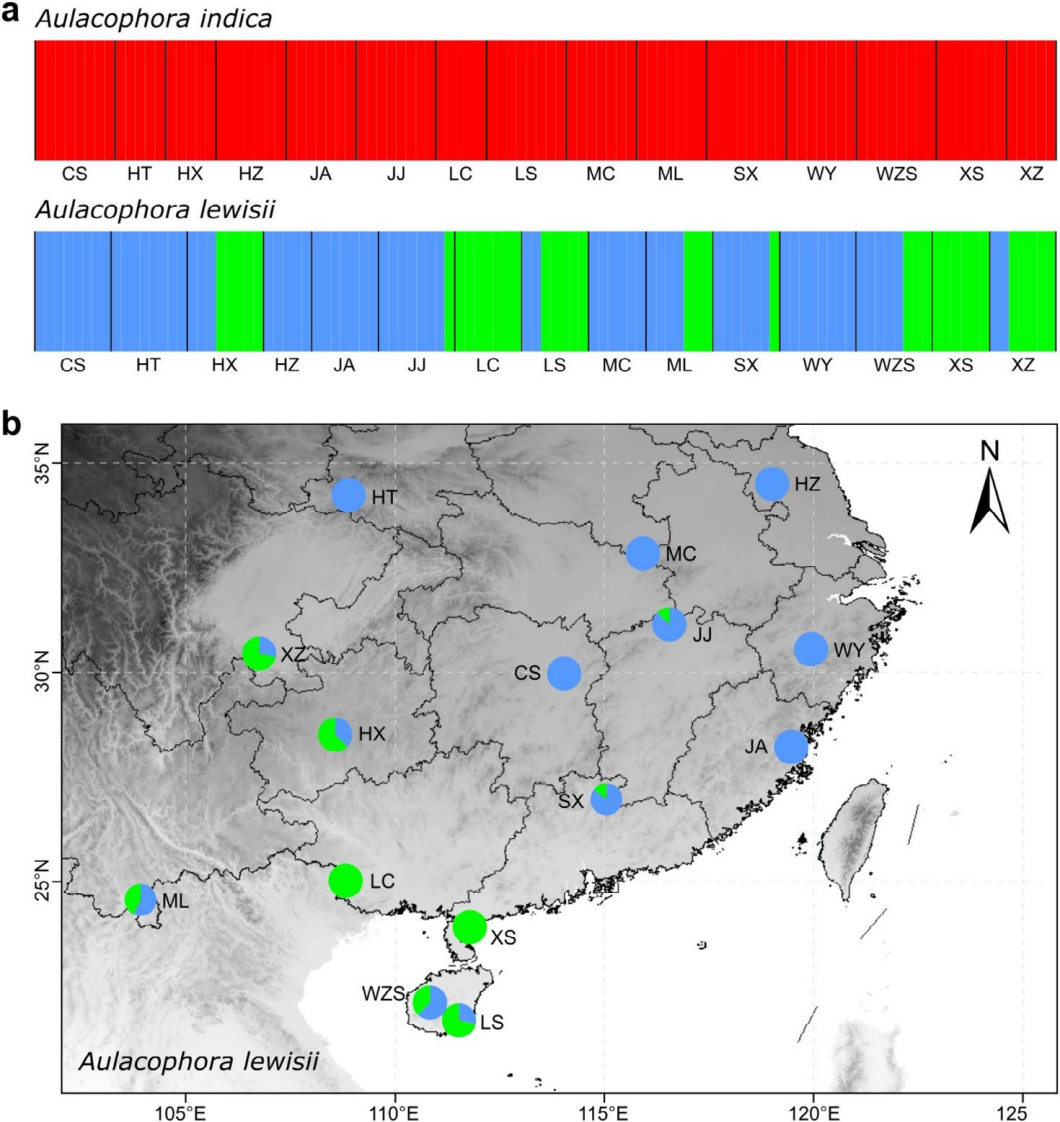
(a) Bayesian analysis of population structure (BAPS) results for *A. indica* (left) and *A. lewisii* (right). *A. indica* populations formed a single genetic cluster, while *A. lewisii* populations showed admixture of two genetic clusters. (b) Geographic distribution of the 15 *A. lewisii* populations with pie charts indicating the proportion of ancestry from the two genetic clusters identified by BAPS.

Adults of two cucurbit leaf beetles ( *A. indica* and *A. lewisii* ) were collected between 2019 and 2020 and 2023 from 15 co‐distributed collection sites in China (Table 1; Figure 2b). All the samples were randomly collected from cucurbit plants planted in farmland. The cultivation of these host plants followed conventional local farming practices, with no application of systemic insecticides that could affect beetle behavior or survival. The 102 *A. indica* samples were collected from cucumber, loofah, pumpkin, bottle gourd, sweet gourd, and white gourd, and 109 *A. lewisii* samples were collected from loofah (Table S1). To avoid the aggregation of siblings, the distance between samples from each collection site was > 50 m. Individuals were stored in absolute ethanol and frozen at −20°C until further laboratory examinations. Species were identified using standard taxonomic keys under a stereomicroscope (Lee and Beenen 2015). We also used *COX1* gene to conduct NCBI online Blast alignment on these samples to ensure the accuracy of species identification (references: Das et al. 2020). Total genomic DNA was extracted from the thorax of each individual using the QIAGEN DNeasy Blood & Tissue Kit (Hilden, Germany), following the manufacturer's protocols.

**TABLE 1:** Collection information for specimens of *A. indica* and *A. lewisii* .

Code (Locality)	Collection date	Longitude (E)	Latitude (N)	Altitude (m)	Number	
					*A. indica*	*A. lewisii*
CS (Changsha, Hunan)	2019/9/15	113.04	28.22	58	8	8
HT (Hantai, Shanxi)	2023/8/10	107.04	33.12	536	5	8
HX (Huaxi, Guizhou)	2023/8/6	106.66	26.52	1105	5	8
HZ (Hongze, Jiangsu)	2023/8/24	118.86	33.19	20	7	5
JA (Jinan, Fujian)	2019/9/2	119.28	26.18	416	7	7
JJ (Jiujiang, Jiangxi)	2023/8/25	115.94	29.57	142	8	8
LC (Longchuan, Guangxi)	2023/7/31	106.96	22.47	194	5	7
LS (Lingshui, Hainan)	2023/7/28	109.94	18.66	79	8	7
MC (Macheng, Hubei)	2019/11/10	115.16	31.53	174	7	6
ML (Mengla, Yunnan)	2023/8/2	101.26	21.93	553	7	7
SX (Shixing, Guangdong)	2020/8/23	114.14	24.69	255	8	7
WY (Wuyi, Zhejiang)	2019/11/1	119.86	28.85	90	7	8
WZS (Wuzhishan, Hainan)	2023/7/25	109.53	18.94	219	8	10
XS (Xiashan, Guangdong)	2023/7/29	110.38	21.18	10	7	6
XZ (Xuzhou, Sichuan)	2023/8/6	104.59	28.73	292	5	7

### 2.2 Sequencing and Mitogenome Data Acquisition

In this study, next‐generation genome sequencing was used to obtain mitochondrial genome sequences. Genomic libraries were constructed, with the insert size of 150 base pairs. The genome resequencing for each sample was performed using the BGI sequencing platform. Quality control for raw sequence data was carried out using fastp v.0.20.0 (Chen et al. 2018). The mitochondrial genomes (mitogenomes) were assembled from whole‐genome sequencing reads using GetOrganelle v.1.7.0 (Jin et al. 2020). In this study, only 13 protein‐coding genes (PCGs) were considered due to the absence of tRNA, rRNA, or control region in the mitochondrial genome assembly results of some individuals. Nucleotide sequences for 13 PCGs were separately translated into amino acids, aligned with MUSCLE implemented within MEGA v.6.05 (Tamura et al. 2013), and then toggled back into nucleotide alignments. Additionally, to eliminate divergent regions and poorly aligned positions, Gblock v.0.91b (Castresana 2000) was employed with default settings. Sequence concatenation was performed using BioEdit v.7.0.9.0 (Hall 1999). The mitogenomes of 13 PCGs and concatenated sequences were deposited in Supporting Information or Zenodo online repository (https://doi.org/10.5281/zenodo.17118799).

### 2.3 Genetic Diversity and Population Structure Analyses

The genetic diversity indices were estimated using DnaSP v.5.10.01 (Librado and Rozas 2009), including the number of haplotypes (*N*), haplotype diversity (*Hd*) and nucleotide diversity (*Pi*). These parameters were calculated for each species based on their 15 geographic populations separately, and also based on all individuals pooled as a single population (i.e., combining all samples of *A. indica* and *A. lewisii* separately). To determine population structure, a Bayesian analysis of population structure (BAPS) model was performed on the concatenated sequence matrix using BAPS v.6.0 (Cheng et al. 2013). To ensure consistency convergence of results, spatial genetic mixture analyses of individuals and populations were performed using a maximum *K* value, each repeated 5 times (Corander et al. 2008; Cheng et al. 2013). In addition, to estimate genetic variation between and within populations of *A. indica* and *A. lewisii* , respectively, we analyzed pairwise *F*_ST_, gene flow (*N*_m_) and differential hierarchical molecular variance (AMOVA) for both species based on concatenated gene sequences using Arlequin v.3.5 (Excoffier and Lischer 2010). Genetic differentiation was regarded as low for *F*_ST_ < 0.05, moderate for 0.05 < *F*_ST_ < 0.15, high for 0.15 < *F*_ST_ < 0.25 and very high for *F*_ST_ > 0.25 (Govindaraju 1989). The existence of patterns of genetic divergence associated with isolation‐by‐distance (IBD) was tested by using correlations between genetic distance and geographic distance matrices. The Mantel test of the geographic distance and genetic distance (*F*_ST_) was performed using the ade4 package in R v.4.3.3 (Dray and Dufour 2007). Gene flow (*N*_m_) was determined using the equation *N*_m_ = 0.5 (1 − *F*_ST_)/*F*_ST_ (Asadollahi et al. 2013; McDermott and McDonald 1993).

### 2.4 Demographic History

Neutrality tests (Tajima's *D* and Fu's *F*s statistics) were performed for each of the 15 populations using DnaSP v.5.10.01 to infer signals of recent demographic changes, such as population expansion or contraction. In neutrality tests, a significantly negative value of Tajima's *D* indicates the presence of positive selection or a sudden population expansion following a recent bottleneck event. A significantly positive value suggests an increase in the average pairwise genetic diversity within the population, indicating a balancing selection model or a population contraction event. Nonsignificant and near zero values of Tajima's *D* imply a constant population size.

### 2.5 Statistical Analysis

Statistical analyses were performed using SPSS v.25.0 (IBM Corp., Armonk, NY, USA). Prior to parametric tests, data normality was assessed for each genetic parameter (*Pi*, *N*_m_, and Tajima's *D*) across all populations using the one‐sample Kolmogorov–Smirnov test (all *p* > 0.05). Paired‐sample *t*‐tests were then used to compare mean values between the two species, with statistical significance set at *p* < 0.05.

## 3 Results

### 3.1 Genetic Diversity

Mitochondrial genome data of 102 *A. indica* and 109 *A. lewisii* samples were generated from 15 geographical populations per species in China, and the concatenated lengths after sequence alignment were 11,076 and 11,196 bp, respectively (Table 2). The comparison of 13 individual PCGs showed that *A. indica* had 14~92 haplotypes (*N*) and 0.397~0.998 haplotype diversity (*Hd*), with the nucleotide diversity (*Pi*) ranging from 0.0036 to 0.0074. *Aulacophora lewisii* had an *N* of 5~59, *Hd* of 0.072~0.969, with *Pi* ranging from 0.0004 to 0.0043 (Table 2). The genes with the greatest and the smallest interspecific difference of *Pi* between *A. indica* and *A. lewisii* were *ND2* (0.0054) and *ATP6* (0.0014), respectively. In concatenated sequence analysis, due to the length of the sequence, the number of haplotypes is inevitably close to the total sample number, so the *N* calculated by the concatenated sequence is not statistically significant, because the total variation of both species is overloaded. Here only *Pi* provides useful information, with the *Pi* of *A. indica* and *A. lewisii* as 0.0056 and 0.0019, respectively (Table 3). Overall, *A. indica* had significantly higher *Pi* than *A. lewisii* with higher genetic diversity (*t* = 9.178, df = 12, *p* < 0.001), whether comparing a single gene or 13 concatenated PCGs (Tables 2 and 3).

**TABLE 2:** Genetic diversity parameters and neutrality test results of the *A. indica* and *A. lewisii* based on each of the 13 PCGs.

Gene	Length (bp)	*N*	*Hd*	*Pi*	Tajima's *D*	Fu's *F*s
*ATP6*	672/672	55/30	0.910/0.878	0.0038/0.0025	−2.547***/−2.104*	−80.145/−30.382
*ATP8*	153/153	14/6	0.397/0.090	0.0037/0.0006	−2.079*/−1.897*	−14.666/−9.382
*COX1*	1542/1641	88/59	0.996/0.959	0.0069/0.0018	−2.185**/−2.348**	−102.343/−82.106
*COX2*	684/684	51/30	0.912/0.841	0.0043/0.0028	−2.461**/−2.480**	−63.222/−26.986
*COX3*	786/786	73/39	0.988/0.911	0.0059/0.0043	−2.240**/−1.807*	−104.155/−30.661
*CYTB*	1137/1137	85/40	0.994/0.836	0.0062/0.0018	−2.340**/−2.342**	−34.145/−46.644
*ND1*	927/948	66/30	0.984/0.792	0.0057/0.0016	−2.245**/−2.327**	−77.370/−32.071
*ND2*	1011/1011	85/38	0.996/0.874	0.0074/0.0020	−2.149*/−2.372**	−114.494/−43.141
*ND3*	351/351	28/14	0.798/0.257	0.0039/0.0009	−2.040*/−2.438**	−31.004/−21.115
*ND4*	1329/1329	87/44	0.995/0.895	0.0047/0.0017	−2.407**/−2.436**	−133.628/−54.578
*ND4L*	279/279	26/5	0.593/0.072	0.0036/0.0004	−2.425**/−2.002*	−32.625/−6.168
*ND5*	1704/1704	92/58	0.998/0.969	0.0057/0.0017	−2.411**/−2.494***	−122.744/−81.208
*ND6*	501/501	38/18	0.812/0.392	0.0045/0.0011	−2.576***/−2.543***	−40.578/−23.321

*Note:* Each numerical result is *A. indica* on the left and *A. lewisii* on the right.

Abbreviations: *Hd*, haplotype diversity; *N*, number of haplotypes; *Pi*, nucleotide diversity.

* *p* < 0.05.

** *p* < 0.01.

*** *p* < 0.001.

**TABLE 3:** Genetic diversity parameters and neutrality test results of the *A. indica* and *A. lewisii* based on different population.

Population	*Pi*	*Hd*	Tajima's *D*	Fu's *F*s
CS	0.0062/0.0018	0.964/1.000	−1.169/−1.397	2.926/−0.890
HT	0.0040/0.0012	1.000/0.929	−0.867/−0.244	1.398/1.506
HX	0.0018/0.0016	1.000/0.857	−1.195/1.064	0.533/3.997
HZ	0.0054/0.0015	1.000/1.000	−0.845/−1.216	0.913/0.293
JA	0.0057/0.0016	1.000/1.000	−1.072/−1.183	0.973/−0.599
JJ	0.0053/0.0017	1.000/0.964	−1.166/−0.916	0.553/0.743
LC	0.0055/0.0012	1.000/1.000	−0.778/−1.541	1.755/−1.132
LS	0.0065/0.0016	1.000/1.000	−1.330/−1.252	0.782/−0.596
MC	0.0053/0.0018	1.000/1.000	−1.435/−0.845	0.878/0.041
ML	0.0056/0.0017	1.000/1.000	−1.068/−0.811	0.938/−0.540
SX	0.0055/0.0018	1.000/0.952	−0.906/−0.025	0.580/1.326
WY	0.0053/0.0017	0.952/1.000	0.303/−1.133	3.204/−1.030
WZS	0.0067/0.0023	1.000/0.978	−1.385/−1.226	0.813/−1.226
XS	0.0057/0.0011	1.000/1.000	−0.965/−0.416	0.968/−0.701
XZ	0.0019/0.0012	1.000/1.000	−1.069/−0.912	0.556/−1.108
All population	0.0056/0.0019	1.000/0.998	−2.422**/−2.546***	−49.024/−33.443

*Note:* Each numerical result is *A. indica* on the left and *A. lewisii* on the right.

Abbreviations: *Hd*, haplotype diversity; *Pi*, nucleotide diversity.

* *p* < 0.05.

** *p* < 0.01.

*** *p* < 0.001.

Analysis of different populations within species showed that the *Pi* of HX and XZ populations in *A. indica* was 0.0018 and 0.0019, significantly lower than that of the other populations (0.0040~0.0067; Table 3). In all *A. lewisii* populations, the *Pi* of HT, LC, XS and XZ populations was lower (0.0011~0.0012), while that of the other 11 populations ranged from 0.0015 to 0.0023. Notably, the highest genetic diversity of both species was detected in the WZS population ( *A. indica* , *Pi* = 0.0067; *A. lewisii* , *Pi* = 0.0023).

### 3.2 Population Structure

BAPS analysis based on concatenated genes showed that all *A. indica* populations were clustered into one cluster without genetic structure (Figure 2a; Table S2). While *A. lewisii* was derived from the genetic mixture of two genetic clusters and was composed of individuals from six plots, including CS, HT, HZ, JA, MC, and WY as the dominant genetic clusters (Figure 2a,b; Table S2). However, LC and XS were assigned to the second cluster, which represents a close relationship and genetic independence between the two populations. HX, JJ, LS, ML, SX, WZS, and XZ individuals exhibited a mixture of these two clusters.

### 3.3 Genetic Differentiation and Gene Flow Between Populations

**FIGURE 3:**
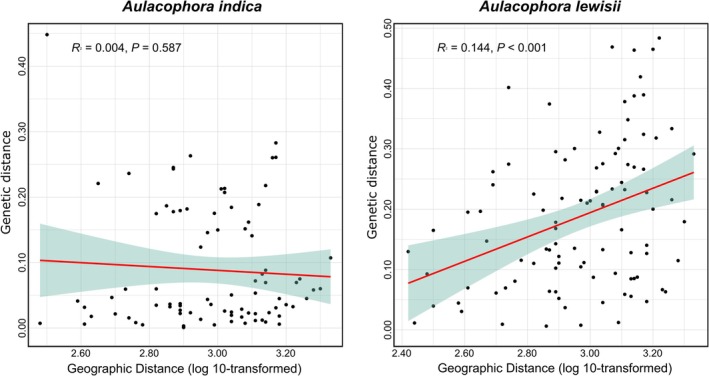
Relationship between gene flow and geographic distance among sites.

The pairwise *F*_ST_ among populations was calculated to quantify their genetic differentiation (Table 4). For *A. indica* , pairwise *F*_ST_ ranged from 0.001 (SX and WZS) to 0.449 (HX and XZ), with an average of 0.089, indicating a low degree of genetic differentiation. For *A. lewisii* , pairwise *F*_ST_ ranged from 0.001 (LS and XS) to 0.484 (HZ and LC), with an average of 0.186, indicating a moderate degree of genetic differentiation. We tested the correlation between genetic differentiation and geographic isolation of the two species. Results showed that there was no significant correlation between *F*_ST_ and geographical distance for *A. indica* (*R* ^2^ = 0.004, *p* = 0.587; Figure 3), indicating that geographical isolation was not their driving force for genetic differentiation. However, the geographical distance and genetic distance of *A. lewisii* showed a significant positive correlation (*R* ^2^ = 0.144, *p* < 0.001; Figure 3), indicating distance isolation effect among these populations. These results indicate that *A. indica* populations are not constrained by geographic distance, whereas *A. lewisii* populations follow an isolation‐by‐distance pattern, reflecting their distinct dispersal and gene flow dynamics.

**TABLE 4:** Pairwise *F*_ST_ results of the *A. indica* (below diagonal) and *A. lewisii* (above diagonal).

	CS	HT	HX	HZ	JA	JJ	LC	LS	MC	ML	SX	WY	WZS	XS	XZ
CS		0.111	0.225	0.178	−0.037	0.040	0.300	0.133	−0.004	0.085	0.070	0.110	0.045	0.282	0.218
HT	0.003		0.374	0.208	0.087	0.215	0.469	0.318	0.102	0.270	0.233	0.232	0.200	0.464	0.402
HX	0.175	0.178		0.388	0.244	0.210	0.196	0.104	0.214	0.132	0.168	0.274	0.135	0.198	0.165
HZ	0.034	0.051	0.218		0.143	0.240	0.484	0.333	0.195	0.292	0.268	0.262	0.216	0.465	0.419
JA	0.035	0.024	0.141	0.037		0.061	0.348	0.166	0.006	0.115	0.069	0.093	0.059	0.328	0.266
JJ	−0.003	−0.009	0.175	−0.007	0.022		0.292	0.128	−0.051	0.067	0.081	0.031	0.056	0.275	0.207
LC	0.013	0.017	0.221	0.033	−0.020	0.007		0.009	0.315	0.115	0.295	0.389	0.147	0.044	0.064
LS	0.020	−0.006	0.146	−0.005	−0.011	−0.013	−0.039		0.140	−0.018	0.122	0.228	0.011	0.001	0.012
MC	0.018	−0.027	0.150	0.006	0.033	−0.011	0.012	−0.020		0.063	0.063	0.000	0.047	0.301	0.229
ML	0.088	0.069	0.245	0.107	0.058	0.070	0.005	0.005	0.075		0.085	0.179	0.008	0.112	0.037
SX	0.032	0.029	0.179	0.026	−0.008	0.008	0.027	0.003	0.022	0.082		0.134	0.052	0.275	0.230
WY	0.060	0.072	0.189	0.047	0.007	0.041	0.031	0.006	0.059	0.060	0.036		0.127	0.378	0.324
WZS	0.024	−0.005	0.124	0.045	0.022	0.012	−0.006	−0.002	0.018	0.044	0.001	0.045		0.130	0.094
XS	0.023	0.009	0.187	0.036	−0.005	0.010	−0.055	−0.018	0.023	0.036	0.016	0.053	−0.005		0.087
XZ	0.182	0.236	0.449	0.260	0.261	0.184	0.243	0.162	0.207	0.263	0.213	0.283	0.152	0.213	

Results based on AMOVA demonstrated that 94.64% of the total molecular variance for *A. indica* was due to intra‐population variation, while the remainder (5.36%) occurred across populations. However, 82.21% of the total molecular variance for *A. lewisii* was due to intra‐population variation, while the remainder (17.79%) occurred across populations. At the population level, the gene flow of the two species was quantified, and the results showed that the total inter‐population mean gene flow (*N*_m_) of *A. indica* was 212.901, while that of *A. lewisii* was 62.860 (Table 5). The paired sample *t*‐test revealed a significant difference in mean gene flow between the two species (*t* = 3.556, df = 104, *p* < 0.001). These results suggested that there was frequent gene flow among the populations of *A. indica* and less among *A. lewisii* .

**TABLE 5:** Gene flow (*N*_m_) results of the *A. indica* (below diagonal) and *A. lewisii* (above diagonal).

	CS	HT	HX	HZ	JA	JJ	LC	LS	MC	ML	SX	WY	WZS	XS	XZ
CS		4.017	1.721	2.304	inf	12.130	1.164	3.261	inf	5.374	6.675	4.037	10.537	1.275	1.794
HT	169.818		0.836	1.907	5.219	1.829	0.567	1.073	4.387	1.354	1.642	1.652	1.998	0.579	0.745
HX	2.360	2.312		0.789	1.547	1.880	2.045	4.286	1.838	3.275	2.476	1.325	3.199	2.021	2.532
HZ	14.113	9.385	1.796		3.003	1.580	0.534	0.999	2.063	1.215	1.362	1.408	1.818	0.575	0.692
JA	13.821	20.774	3.047	12.947		7.682	0.936	2.514	79.632	3.856	6.699	4.890	7.995	1.026	1.378
JJ	inf	inf	2.352	inf	22.638		1.211	3.407	inf	7.004	5.696	15.881	8.496	1.316	1.920
LC	37.044	29.167	1.763	14.807	inf	67.560		53.151	1.086	3.833	1.193	0.784	2.899	10.740	7.323
LS	25.027	inf	2.930	inf	inf	inf	inf		3.066	inf	3.608	1.697	43.188	992.320	40.342
MC	27.574	inf	2.837	82.085	14.879	inf	42.982	inf		7.421	7.420	inf	10.136	1.163	1.686
ML	5.159	6.696	1.539	4.162	8.082	6.685	99.864	102.109	6.162		5.409	2.289	64.274	3.983	13.067
SX	15.259	16.832	2.286	18.740	inf	59.310	17.831	156.042	21.969	5.566		3.232	9.088	1.321	1.677
WY	7.856	6.440	2.150	10.197	68.317	11.604	15.676	83.247	7.911	7.821	13.207		3.448	0.822	1.043
WZS	19.960	inf	3.542	10.556	22.271	40.800	inf	inf	26.785	10.934	557.037	10.605		3.354	4.835
XS	20.827	53.516	2.178	13.494	inf	49.579	inf	inf	21.191	13.361	31.564	8.866	inf		5.238
XZ	2.248	1.616	0.614	1.421	1.417	2.212	1.556	2.590	1.914	1.400	1.844	1.268	2.797	1.852	

*Note:* inf, high‐level gene flow (infinite).

### 3.4 Demographic History

Neutrality tests were conducted using Tajima's *D* and Fu's *F*s statistics. When all populations were considered as a whole, significant negative values were detected among *A. indica* (Tajima's *D* = −2.422, *p* < 0.01; Fu's *F*s = −49.024) and also among *A. lewisii* (Tajima's *D* = −2.546, *p* < 0.001; Fu's *F*s = −33.443; Table 3). Furthermore, the Tajima's D values for each PCG were significant (*p* < 0.05; Table 2), and Fu's *F*s values were also negative. These results are consistent with either recent population expansion or the action of positive selection in both species. At the population level, we detected negative Tajima's *D* values in all populations except WY of *A. indica* and HX of *A. lewisii* , but these values were not significant (*p* > 0.05; Table 3), likely due to the reduced statistical power of these tests with small sample sizes per site. For most populations, the neutrality test results did not deviate significantly from neutral expectations, suggesting that random genetic drift may play a predominant role in shaping genetic variation within populations.

## 4 Discussion

*Aulacophora indica* and *A. lewisii* are very common pests of cucurbit crops, usually occurring in large numbers, thereby significantly reducing crop yields (Kamal et al. 2014; Lee and Beenen 2015). Therefore, elucidations of the potential evolutionary patterns and population adaptations of these two *Aulacophora* species are crucial for pest control. In this study, mitochondrial genome‐based analyses revealed markedly higher genetic diversity in *A. indica* compared to *A. lewisii* , indicating that *A. indica* possesses stronger gene exchange capability among populations and greater environmental adaptability. This disparity in genetic diversity between the two species suggests that dietary breadth may play a key role in shaping their population genetic characteristics.

Genetic variation is essential for species to survive and adapt to different environmental conditions (Hofinger et al. 2011; Pérez‐Alquicira et al. 2019; Scheffer and Lewis 2006; Wang et al. 2021; Ye et al. 2022). In this study, the AMOVA analysis revealed that, compared to *A. indica* , *A. lewisii* exhibits a higher degree of genetic variation among (17.79%) populations, along with significant genetic differentiation (*F*_ST_ = 0.186) and low gene flow (*N*_m_ = 62.860). This could be attributed to physical barriers (such as mountains and fragmented habitats) or other critical factors (like flight activity and host plant availability) that restrict gene flow (Miyakawa et al. 2018; Xue et al. 2014). *Aulacophora indica* and *A. lewisii* are small‐sized insects with moderate flight and dispersal capabilities; thus, they exhibit only short‐distance movements. In general, the isolation by distance (IBD) effect is pronounced in species with moderate mobility but weaker in those with low or high mobility (Peterson and Denno 1998). Our analysis revealed different geographic dispersal patterns between the two species; that is, *A. indica* showed no significant relationship between population genetic differentiation and IBD (*R* ^2^ = 0.004, *p* = 0.587), indicating its free migration is unaffected by physical barriers. In contrast, *A. lewisii* was influenced by IBD (*R* ^2^ = 0.144, *p* < 0.001). Studies on the genetic diversity and phylogeography of highly migratory species have found that geographic barriers are the primary factor driving differentiation (Fan et al. 2014; Meng et al. 2008; Ohwaki et al. 2018). Conversely, for some Coleoptera species with weak flight capabilities, natural geographic factors and host plants often play a crucial role in shaping the genetic structure and gene flow among populations (Nakadai and Kawakita 2016; Schluter 2009; Toju and Sota 2006; Xue et al. 2010, 2014). During the sampling process, we did not observe significant differences in flight capability between *A. indica* and *A. lewisii* . Additionally, the geographic distribution of the sampled populations of both species in this study overlaps, and they are subject to similar natural geographic factors. Therefore, we hypothesize that the observed genetic differences may be caused by factors other than physical barriers and migration ability, such as host plant specificity, dietary breadth, and human‐mediated crop cultivation practices, which may differentially influence population connectivity and gene flow between the two species.

Herbivorous insects and their host plants have developed a mutually adaptive relationship through a long process of co‐evolution for survival and reproduction. The phenology, geographic distribution, and ecological isolation of host plants can influence the habits, geographic variation, and genetic differentiation of insects (Hood et al. 2020; Kébé et al. 2017). Host plant specialization is a key mechanism driving the diversification of herbivorous insects (Drummond et al. 2009; Jaenike 1990). *Aulacophora* beetles primarily feed on cucurbit plants (Lewis and Metcalf 1996), and they are particularly attracted to the cucurbitacin compounds found in these plants, which has led to their specialization as cucurbit‐feeding insects (Abe and Matsuda 2005; Kong et al. 2004). Genetic differentiation is more likely to occur among insect populations that utilize chemically distinct host species, especially for oligophagous beetle with limited dispersal capabilities (Kelley et al. 2000). Although both *A. indica* and *A. lewisii* are specialized to feed on cucurbit plants, the former has a wider range of dietary habits and inflicts damage on a greater variety of cucurbit crops (Ahmad et al. 2013; Kamal et al. 2014; Lee and Beenen 2015). In contrast, *A. lewisii* is an oligophagous pest that feeds exclusively on loofah (see Table S1). Notably, these cucurbit crops are widely and sporadically cultivated in rural areas of southern China, often found in small vegetable plots around villages or in scattered landscapes near houses (Figure 1c,d). The polyphagous behavior of *A. indica* allows it to move and disperse among a wider variety of host plants than the oligophagous *A. lewisii* , which may lead to greater population connectivity and consequently differences in gene flow between the two species. Studies have shown that the genetic differentiation of specialized herbivorous beetles is significantly influenced by their host plants (Conedera et al. 2004; Mynhardt et al. 2007; Zhang et al. 2024). When host plants migrate or their distribution changes, the long‐established population distribution patterns of the insects are disrupted, leading to corresponding changes in their genetic structure (Conedera et al. 2004; Mynhardt et al. 2007; Zhang et al. 2024). Therefore, we hypothesize that the oligophagous nature of *A. lewisii* makes its populations more susceptible to constraints in gene flow, resulting in higher genetic differentiation.

For many pests with limited flight capabilities, human‐mediated crop cultivation, particularly plant propagation activities and commercial trade, can provide potential pathways for high genetic exchange (Avtzis and Cognato 2013; Bonal et al. 2016; Hood et al. 2020; Zhang et al. 2018). In this study, neutrality tests revealed significantly negative Tajima's *D* and the Fu's *F*s values in both *Aulacophora* species, which may reflect either recent population expansion or the action of positive selection. In China, cucurbit crops have become widely integrated into household cultivation across most regions. The patchwork of vegetable gardens cultivates a modest number of cucurbit plants, providing favorable conditions for the expansion of cucumber beetle populations, particularly the dispersal of *A. indica* populations facilitated by the cultivation of their host plants. Therefore, we hypothesize that the observed genetic differences between the two species are closely associated with their fundamental dietary differences (polyphagous in *A. indica* vs. oligophagous in *A. lewisii* ) and the consequent disparity in the spatial distribution of their host plants, which has been further amplified by human‐mediated crop cultivation and landscape modification. However, further biologically relevant studies are needed to confirm the differences attributable to this factor.

## Author Contributions

**Bo He:** funding acquisition (equal), methodology (equal), software (equal), writing – original draft (equal). **Mingkang Xiao:** methodology (equal), writing – original draft (supporting). **Tianyu Ye:** methodology (equal), resources (equal). **Gonghua Lin:** methodology (equal), software (equal). **Fang Zhao:** resources (equal). **Liancheng Liu:** resources (equal). **Huanhuan Li:** resources (supporting). **Tianjuan Su:** conceptualization (equal), resources (equal), writing – original draft (supporting). **Zuhao Huang:** conceptualization (equal), funding acquisition (equal), resources (equal).

## Funding

The research was supported by the National Natural Science Foundation of China (32460304, 32100336), the Natural Science Foundation of Jiangxi Province (20212ACB205006, 20252BAC200373), the Jiangxi “Double Thousand Plan” (jxsq2023201063).

## Conflicts of Interest

The authors declare no conflicts of interest.

## Supporting information


**Table S1:** Collection and host plant information for specimens of *A. indica* and *A. lewisii* .

**Table S2:** List of sizes of 10 best visited partitions and corresponding log (ML) values.

## Data Availability

The datasets generated and analyzed during the current study are available in the Zenodo repository, https://doi.org/10.5281/zenodo.17118799.
